# SH3GL1‐activated FTH1 inhibits ferroptosis and confers doxorubicin resistance in diffuse large B‐cell lymphoma

**DOI:** 10.1002/ctm2.70246

**Published:** 2025-03-04

**Authors:** Zi‐Wen Duan, Wei‐Ting Wang, Yan Wang, Rong Wang, Wei Hua, Chun‐Yu Shang, Rui Gao, Hao‐Rui Shen, Yue Li, Jia‐Zhu Wu, Hua Yin, Li Wang, Jian‐Yong Li, Wei Xu, Jin‐Hua Liang

**Affiliations:** ^1^ Department of Hematology the First Affiliated Hospital of Nanjing Medical University, Jiangsu Province Hospital Nanjing China; ^2^ Key Laboratory of Hematology of Nanjing Medical University Nanjing China; ^3^ Collaborative Innovation Center for Cancer Personalized Medicine Nanjing China; ^4^ Department of Endocrinology the First Affiliated Hospital of Nanjing Medical University, Jiangsu Province Hospital Nanjing China

**Keywords:** diffuse large B‐cell lymphoma, doxorubicin resistance, ferritinophagy, ferroptosis, FTH1, SH3GL1

## Abstract

**Background:**

Diffuse large B‐cell lymphoma (DLBCL) is predominant subtype of non‐Hodgkin lymphoma and can be effectively treated. Nevertheless, a subset of patients experiences refractory or relapsed disease, highlighting the need for new therapeutic strategies.

**Methods:**

Depmap database based on CRISPR/Cas9 knock out analysis was employed to identify the essential gene SH3GL1, which encodes endophilin A2, as crucial for the proliferation and survival of DLBCL cells. Immunohistochemistry (IHC) staining was performed on the 126 paraffin‐embedded clinical DLBCL samples to investigate the association between SH3GL1 expression levels and the prognosis. To investigate the specific mechanism modulated by SH3GL1 in the progression of DLBCL, an integrative approach was employed. This approach combined high‐throughput sequencing technologies, such as Deep‐DIA and LC‐MS, with functional validation techniques, including CRISPR/Cas9 gene editing, xenograft models, and molecular pathway analyses.

**Results:**

Our study found that high expression levels of SH3GL1 correlate with poor prognosis in a cohort of 126 newly diagnosed DLBCL patients, underscoring its significance in disease progression. Mechanistically, we found that SH3GL1 deficiency triggers ferritin heavy chain 1 (FTH1)‐mediated ferroptosis, specifically ferritinophagy‐induced ferroptosis, in DLBCL cells. Additionally, high expression of SH3GL1 suppresses doxorubicin‐induced ferroptosis. Cancer cells' resistance to conventional therapies is associated with increased sensitivity to ferroptosis.

**Conclusions:**

These findings emphasise SH3GL1 as a promising prognostic biomarker and a potential therapeutic target in DLBCL, offering new avenues for treatment strategies aimed at overcoming drug resistance and improving patients' outcomes.

**Key points:**

- Elevated SH3GL1 expression in DLBCL patients was associated with a negative prognosis.
- SH3GL1 plays a crucial role in promoting DLBCL cell survival through the regulation of FTH1‐mediated ferroptosis and doxorubicin resistance.

## Graphical abstract

Elevated expression levels of SH3GL1 in patients with diffuse large B‐cell lymphoma (DLBCL) was associated with poor prognostic outcomes.

SH3GL1 plays an important role in promoting DLBCL cell survival through the regulation of ferritin heavy chain 1 (FTH1)‐mediated ferroptosis and doxorubicin resistance.

SH3GL1 can be a valuable prognostic biomarker and a potential target for anti‐DLBCL intervention in the future

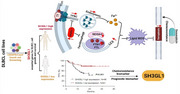

## 1 INTRODUCTION

Diffuse large B‐cell lymphoma (DLBCL) is the predominate non‐Hodgkin lymphoma subtype, characterised by aggressive, biological heterogeneous and poor prognosis. Despite progress in chemotherapy, particularly with rituximab (anti‐CD20 antibody) have improved the survival rates among patients with DLBCL, yet 40%–50% of patients suffer from disease progression.^1^ The heterogeneity of DLBCL genetic subtypes and resistance to apoptosis remain major challenges for current treatment.^2^, ^3^, ^4^, ^5^ Therefore, there is an urgent need and significant clinical value to explore new therapeutic targets and discover novel therapeutic strategies.

CRISPR/Cas9 technology has been widely used to identify genes, which were essential for proliferation and survival of cancer cells. Depmap database (https://depmap.org/portal/) incorporates results from genome‐wide CRISPR/Cas9 loss‐of‐function screens on over 1000 cancer cell lines. This database has greatly benefited researchers to identify potential targets for cancer treatment.^6^ Here, by delving into the Depmap database, we revealed that SH3GL1 is an essential gene in DLBCL cells. SH3GL1 mainly encodes endophilin A2, which is closely associated with cellular endocytosis. Additionally, SH3GL1 is implicated in vesicle endocytosis, signal transduction, mitochondrial metabolism, autophagy and various other mechanisms, with potential implications for the pathogenesis of multiple diseases, making it an attractive target and a potential biomarker.^7^, ^8^, ^9^, ^10^, ^11^ To date, there is limited studies investigating the role of SH3GL1 in the pathogenesis of DLBCL, and more research is needed.

Ferroptosis is a newly identified iron‐dependent type of non‐apoptotic cell death, which was distinguished by elevated levels of reactive oxygen species (ROS) and excessive lipid peroxidation. Ferroptosis is critically involved in the molecular pathogenesis of many cancers.^12^ Additionally, cancer cells that develop resistance to conventional therapies have been shown to have increased sensitivity to ferroptosis, suggesting targeting ferroptosis has remarkable potential in cancer treatment.^13^, ^14^ Although ferroptosis is involved in the tumourigenesis, progression and drug resistance of DLBCL, the detailed role remains unclear.^15^, ^16^, ^17^, ^18^ The ferritin heavy chain 1 (FTH1) is essential for maintaining cellular iron homeostasis in ferroptosis, which is intricately regulated by the iron metabolism.^19^ FTH1 is important in ferritinophagy, which is a selective type of autophagy. Ferritinophagy refers to the autophagic degradation of ferritin, leading to elevated intracellular iron levels. Recent research indicates that ferritinophagy‐induced ferroptosis may contribute to cancer progression, including DLBCL, although the precise mechanisms involved require further investigation.^20^, ^21^, ^22^, ^23^

Our research aims to investigate the function of SH3GL1 in DLBCL pathogenesis. We found that SH3GL1 is the key regulator that inhibits ferroptosis‐induced cell death via FTH1 in DLBCL cells. This study highlights SH3GL1 as a novel regulator of ferroptosis in DLBCL, suggesting that targeting SH3GL1 could be a promising strategy to prevent DLBCL progression and overcome doxorubicin resistance.

## 2 METHODS

### 2.1 Materials and methods

More detailed information is available in the Supporting Information Materials and Methods section.

### 2.2 Patients

Primary tumour samples from 126 patients with DLBCL were collected from the First Affiliated Hospital of Nanjing Medical University with informed consent and approval from ethics commission. All samples were collected prior to first‐line regimen. Clinical features of patients with DLBCL, such as age, gender, Eastern Cooperative Oncology Group (ECOG), lactate dehydrogenase (LDH), extranodal sites, were collected for analysis. All patients received R‐CHOP regimen, which comprises rituximab, cyclophosphamide, doxorubicin, vincristine and prednisone, as their first‐line treatment regimen.

### 2.3 Cell cultures and growth curves

DLBCL cell lines including FARAGE, SUDHL4 and SUDHL8 were acquired from the American Tissue Culture Collection (ATCC), while BJAB and KIS‐1 cell lines were obtained from the Deutsche Sammlung von Mikroorganismen und Zellkulturen (DSMZ). Cell lines were cultured in Roswell Park Memorial Institute (RPMI) 1640 medium (Gibco, C11875500BT) with 10% foetal bovine serum (FBS; VivaCell, C04001‐500). HEK293T cell line was obtained from the BeNa Culture Collection and cultured in DMEM (Gibco, 11995065) with 10% FBS. All cell lines were incubated in a humidified incubator with 5% CO_2_ at 37°C. Cell counting was conducted using a cytometer (Countstar BioTech), and cellular growth was subsequently analysed.

### 2.4 CRISPR/Cas9 gene editing

Knockout of genes was achieved using the CRISPR/Cas9 system, as outlined in a previous study.^24^ The Benchling CRISPR tool was used to design two gene‐specific sgRNAs for each target gene. Lentiviruses expressing sgRNAs were obtained through transfection of HEK293T cells. Additional information on the experimental procedures can be found in the Supporting Information Materials and Methods section.

### 2.5 In vivo xenograft study

All animal experiments were performed in compliance with guidelines established by the Institutional Animal Care and Use Committee of Nanjing Medical University (Ethics Approval Number: IACUC‐2310063), adhering to the regulations set by the National Institutes of Health (NIH). The construction of FARAGE and SUDHL8 subcutaneous mouse models followed previously published methods.^25^

### 2.6 Western blotting, immunohistochemistry and immunohistochemistry analysis

Western blotting (WB), immunohistochemistry (IHC) and immunohistochemistry (IF) analysis were conducted as previously outlined.^25^, ^26^ Antibodies used were detailed in Table S1. ZEISS microscopy was used to image the stained sections of IHC. SH3GL1 scoring was visually assessed in 10% increments. Tumour cells with over 30% SH3GL1 expression were marked positive (+), while those below this threshold were classified as negative (−). Positive cases were further categorised by staining intensity from + to ++++, with scores independently evaluated by two pathologists.

### 2.7 Statistical analysis

Statistical analyses were performed using GraphPad Prism version 9.0, SPSS version 26.0 and R 4.2.0 software version. Progression‐free survival (PFS) was defined as duration from diagnosis to disease progression or relapse. Overall survival (OS) was determined from diagnosis to death or last follow‐up visit. Progression disease (PD) was assessed according to the Lugano Revised Criteria for Response Assessment.^27^ Kaplan–Meier survival curves were used to estimate PFS and OS, with groups comparisons conducted using the log‐rank test. Cox regression analysis was applied to identify risk factors associated with OS and PFS. Statistical significance was established at as a *p* value <.05.

## 3 RESULTS

### 3.1 SH3GL1 promotes DLBCL cell proliferation in vitro and tumour formation in vivo

**FIGURE 1:**
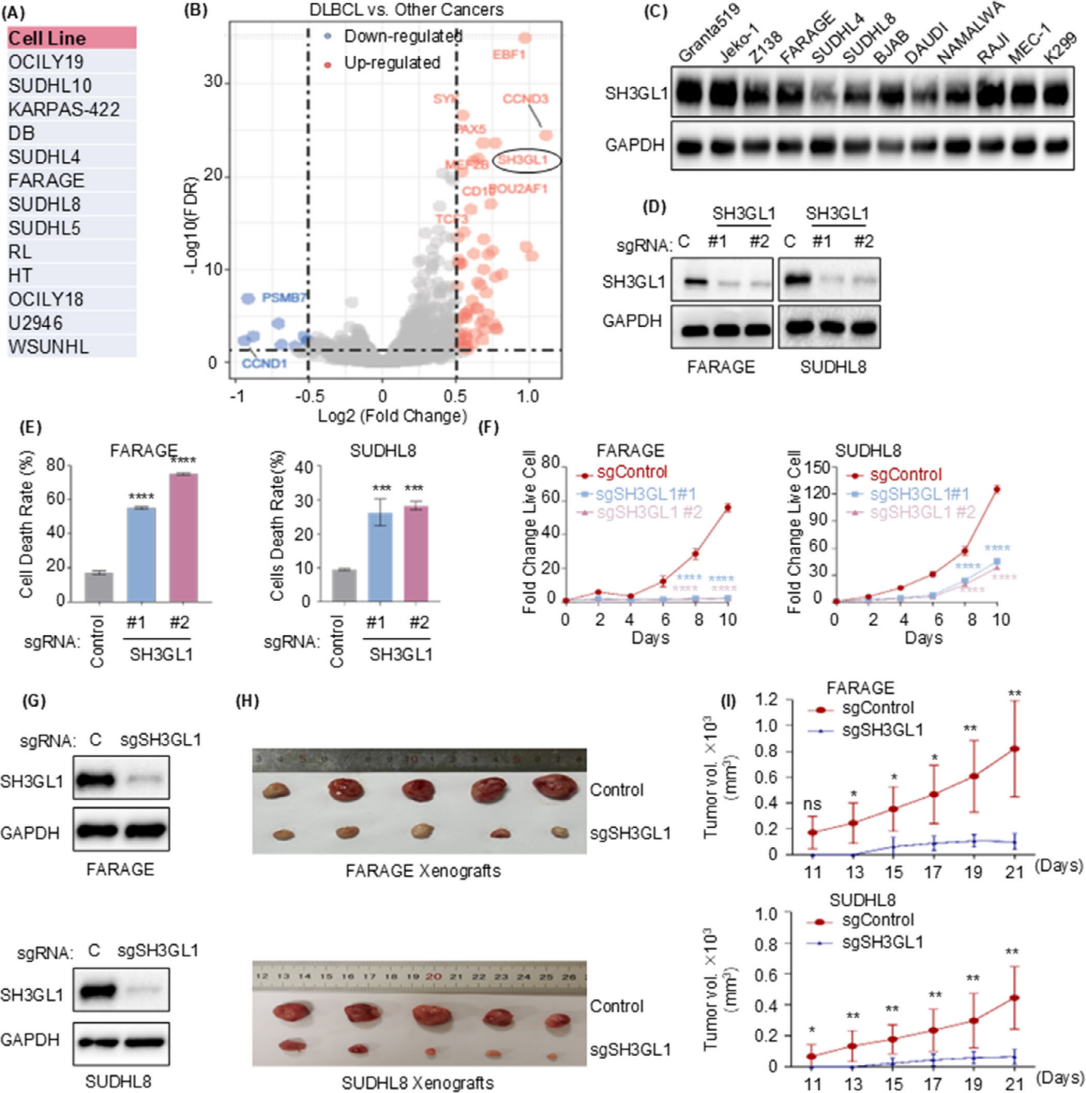
SH3GL1 promotes diffuse large B‐cell lymphoma (DLBCL) cell proliferation and in vivo tumour formation. (A, B) Depmap database showed SH3GL1 is an essential gene in non‐Hodgkin lymphoma cell lines compared with other cancer cell lines (https://depmap.org/portal/). (C) Immunoblot analysis showed high expression of SH3GL1 protein in mantle cell lymphoma (MCL), DLBCL, Burkitt's lymphoma (BL), chronic leukaemia (CLL) and anaplastic large cell lymphoma (ALCL) cell lines. (D) Immunoblot analysis showed the efficacy of SH3GL1 knockout in Cas9+ FARAGE and Cas9+ SUDHL8 expressing non‐targeting control (C) or sgRNA targeting SH3GL1 expression. (E) Flow cytometry analysis of cell death was measured using Propidium iodide (PI) Kit in FARAGE and SUDHL8 at day 7 postexpression of control or sgRNAs targeting SH3GL1. (F) Growth curve analysis of FARAGE and SUDHL8 expressing control or SH3GL1 sgRNAs was performed at day 5 post‐sgRNA expression. (G) The efficacy of SH3GL1 knockout in FARAGE and SUDHL8 xenograft mouse was confirmed by immunoblot analysis. (H, I) Volume analysis (H) and growth curve analysis (I) of tumour in SH3GL1 wild‐type and SH3GL1 knockout group in FARAGE and SUDHL8 xenograft mouse model (*n* = 5 per group). Data are shown as the mean ± SD. * *p* < .05; ** *p* < .01; *** *p* < .001; **** *p *< .0001 using one‐way analysis of variance (ANOVA) with multiple comparisons.

To identify potential targets for DLBCL treatment, genes essential for DLBCL cell survival were investigated from DepMap. There are 13 DLBCL cell lines in this database, as presented in Figure 1A. We used the R package ‘Limma’ within RStudio, and results showed that SH3GL1 is an essential gene for DLBCL cell survival (Figure 1B) compared to other cancer cell lines. Western blotting analysis demonstrated that the levels of SH3GL1 protein were elevated in DLBCL cells (BJAB, FARAGE, KIS‐1, SUDHL8, SUDHL4, CTB1, MEDB1, RIVA, SUDHL2 and U2932) relative to CD19+ B cells isolated from normal human peripheral blood mononuclear cells (Figure S1A). Furthermore, SH3GL1 exhibited high expression levels across mantle cell lymphoma (MCL), Burkitt's lymphoma, chronic leukaemia (CLL), anaplastic large cell lymphoma (ALCL) and DLBCL cell lines (Granta519, Jeko‐1, Z138, FARAGE, SUDHL4, SUDHL8, BJAB, DAUDI, NAMALWA, RAJI, MEC‐1 and KARPARS299; Figure 1C). CRISPR/Cas9 was performed to knock out SH3GL1 to confirm the role of SH3GL1 in DLBCL cell growth and survival. CRISPR/Cas9 efficiency depleted SH3GL1 from each DLBCL cell lines as shown by Western blotting (Figures 1D and S1B). The knockout of SH3GL1 significantly increased DLBCL cell death (Figures 1E and S1C) and inhibited DLBCL cell proliferation (Figures 1F and S1D). However, depletion of SH3GL1 in MEC‐1 and KARPARS299, a CLL cell and ALCL cell line, did not affect their proliferation (Figure S1E,F), suggesting the specificity of SH3GL1 for DLBCL survivals.

To further examine the roles of SH3GL1 in DLBCL in vivo tumour formation, xenograft animal experiment was performed. DLBCL cell line FARAGE and SUDHL8 stably expressing Cas9 were introduced with non‐target control sgRNA or sgRNA targeting SH3GL1 and then subcutaneous injected into xenograft mouse to construct DLBCL xenograft mouse model, with five mice allocated to each group. As shown in Figure 1G–I, a significant decrease of tumour volume was observed in the absence of SH3GL1 compared to SH3GL1 wild‐type group, along with decreased SH3GL1 protein level in SH3GL1 knock out tumours.

Taken together, these results suggested that SH3GL1 is integral to both in vitro cell proliferation and in vivo tumour formation.

### 3.2 High SH3GL1 expressions correlates with poor prognosis in DLBCL patients

**FIGURE 2:**
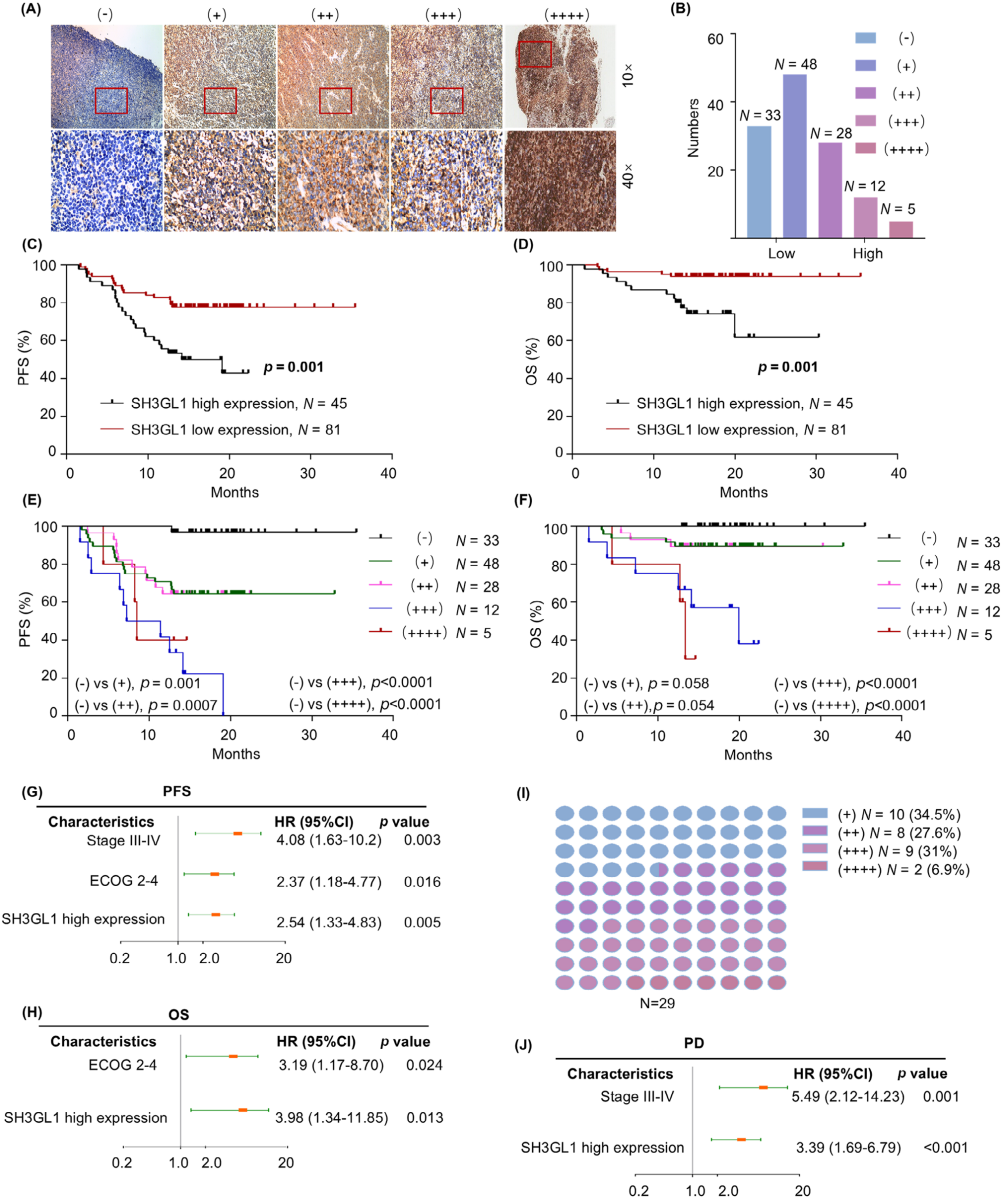
SH3GL1 is overexpressed in human diffuse large B‐cell lymphoma (DLBCL) and associated with prognosis. (A) Representative images of different levels of SH3GL1 expression in DLBCL tumour tissues by immunohistochemistry was shown; *n* = 126. (B) Distribution of SH3GL1 staining by different expression levels. (C, D) Progression‐free survival (PFS) and overall survival (OS) of DLBCL patients based on SH3GL1 expression in 126 DLBCL tumour tissues. (E, F) PFS and OS of DLBCL patients with different expression levels of SH3GL1 in 126 DLBCL tumour tissues. (G, H) Multivariate Cox analysis of PFS and OS for 126 DLBCL patients. (I) Distribution of different levels of SH3GL1 expression in DLBCL patients with progression disease (PD). (J) Multivariate Cox analysis of PD for 126 DLBCL patients. Data are shown as the mean ± SD. * *p* < .05; ** *p* < .01; *** *p* < .001; **** *p *< .0001 using one‐way analysis of variance (ANOVA) with multiple comparisons.

In the study, 126 patients with confirmed DLBCL diagnosis between February 2021 and December 2022 were included. IHC staining was performed on the 126 paraffin‐embedded clinical DLBCL samples, and representative images of different levels of SH3GL1 expression were presented in Figure 2A. In DLBCL patients, 81 patients were divided into SH3GL1 low expression (26.2% with (−) and 38.1% with (+) expression), and 45 patients were grouped into high expression (22.2% with (++) expression, 9.5% with (+++) expression and 4.0% with (++++) expression) by IHC. The distribution was shown in Figure 2B. The clinical features of patients between high expression and low expression of SH3GL1 were presented in Table 1. The results revealed that the ECOG 2–4 and PD status was different in low and high SH3GL1 expression level group. No statically differences were found in any clinical parameters including age, gender, B symptoms, disease stage, LDH, extranodal sites and Hans classification. The analysis of Kaplan–Meier revealed that DLBCL patients with high SH3GL1 expression had poor OS (61.79% vs. 93.83%, *p *= .001) and PFS (42.86% vs. 77.68%, *p *= .002; Figure 2C,D). In addition, we analysed the prognosis of patients with different expression level of SH3GL1, and results showed that the higher the expression level of SH3GL1, the worse the prognosis for patients with DLBCL (Figure 2E,F). Kaplan–Meier analysis from GSE 10846 and GSE 32918 databases also revealed that high SH3GL1 expression was associated with poor OS (34.63% vs. 51.16%, *p *= .006 and 39.28% vs. 67.344%, *p *= .01; Figure S1G,H). Risk factors affecting OS and PFS of DLBCL patients were analysed based on multivariate analysis. The results showed that high SH3GL1 expression (Hazard Ration [HR] 2.54, 95% confidence interval [CI], 1.33–4.83, *p *= .005; HR 3.98, 95% CI, 1.34–11.85, *p *= .013), were independent parameters (Figure 2G,H) of PFS and OS for DLBCL patients.

**TABLE 1:** Correction between SH3GL1 protein expression and characteristics of diffuse large B‐cell lymphoma (DLBCL) patients from Jiangsu Provincial Hospital DLBCL (JSPHDLBCL) database.

		SH3GL1 expression		
	No.	Low (*n* = 81)	High (*n* = 45)	*p* value
Age—years				.34
>60 years	49	29	20	
≤60 years	77	52	25	
Gender				.331
Male	74	45	29	
Female	52	36	16	
IPI score				.14
0–2	78	54	24	
3–5	48	27	21	
Disease stage				.829
I–II	52	34	18	
III–IV	74	47	27	
ECOG PS				**.007**
≤1	107	74	33	
>1	19	7	12	
Extranodal site(s)				.662
1 or 0	78	49	29	
>1	48	32	16	
LDH				.11
≤ULN (270 µmol/)	68	48	20	
>ULN (270 µmol/L)	58	33	25	
B symptom				.217
Yes	47	27	20	
No	79	54	25	
Hans classification				.59
GCB	41	25	16	
Non‐GCB	85	56	29	
LymphGen genotyping				
MCD	34	19	15	.723
A53	20	14	6	
N1	1	1	0	
BN2	21	13	8	
ST2	9	5	4	
EZB	7	4	3	
Other	34	25	9	
PD				**<.001**
Yes	29	10	19	
No	97	71	26	

Abbreviations: ECOG PS, Eastern Cooperative Oncology Group performance status; GCB, germinal centre B‐cell; IPI, International Prognostic Index; LDH, lactate dehydrogenase; PD, Progression Disease; ULN, upper limit of normal.

With the median follow‐up of 17.7 months (1.5–35.5 months), among the 126 DLBCL patients, 29 patients suffered from PD. Among the PD patients, 65.5% exhibited high expression of SH3GL1 (Figure 2I). Furthermore, multivariate analysis was conducted to identify risk factors associated with PD in DLBCL patients (Figure 2J). Results revealed that elevated SH3GL1 expression (HR 3.39, 95% CI, 1.69–6.79, *p *< .001) was an independent factor of patients of PD in these patients. This finding may have implications for the poorer prognosis in DLBCL patients with high SH3GL1 expression.

Collectively, these findings demonstrated that SH3GL1 is clinically important for the survivals in DLBCL patients and could potentially serve as a novel prognostic marker.

### 3.3 Positive correlation between SH3GL1 and ferroptosis signalling in DLBCL cells

**FIGURE 3:**
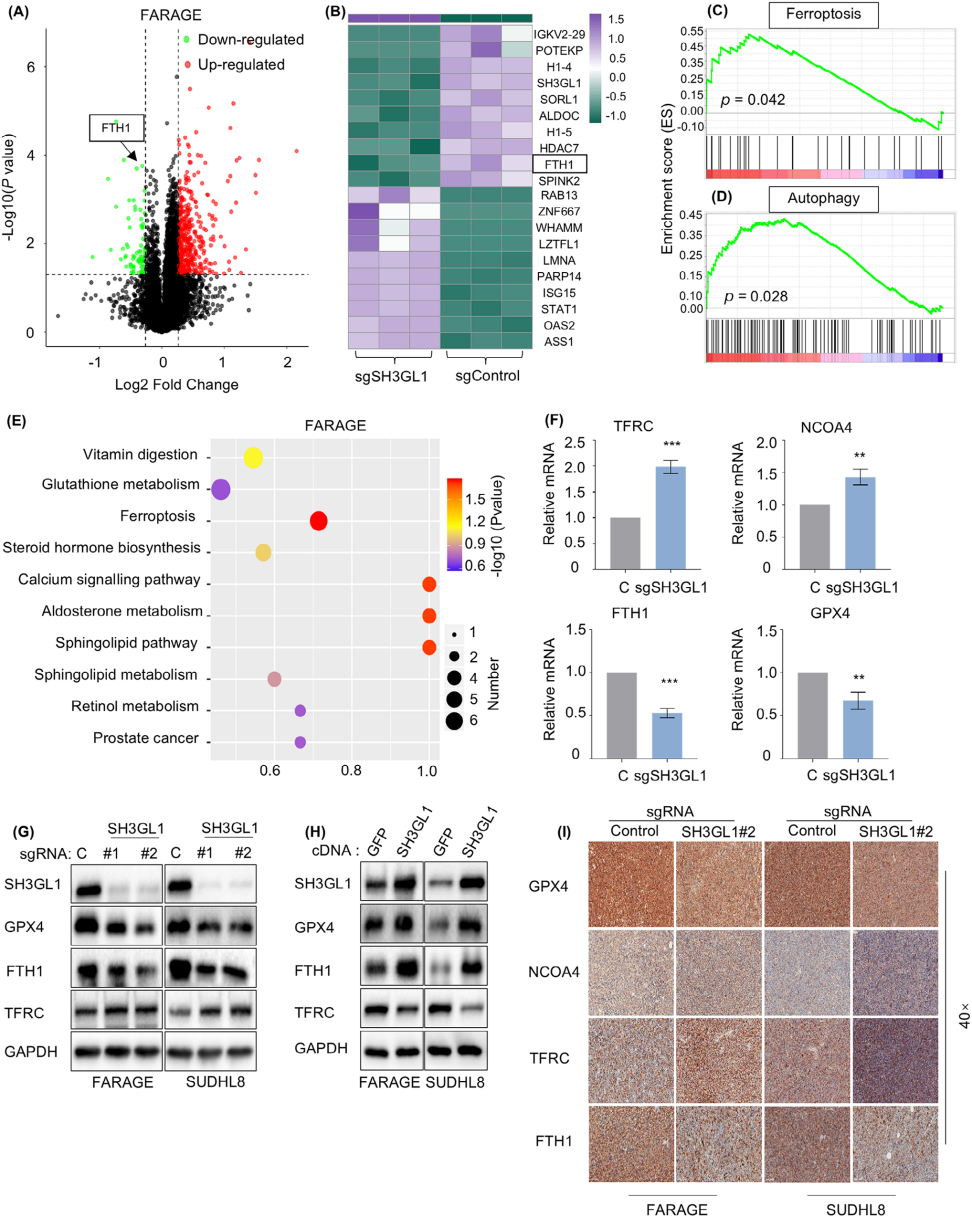
Positive correlation between SH3GL1 and ferroptosis signalling in diffuse large B‐cell lymphoma (DLBCL) cells. (A) Volcano plot of downregulated and upregulated gene sets enriched in FARAGE expressing control or SH3GL1 sgRNAs by Deep Data‐independent acquisition (Deep‐DIA). (B) Unsupervised hierarchical clustering heatmap of top 10 upregulated and top 10 downregulated proteins enriched in Cas9+ FARAGE that express control or SH3GL1 sgRNAs by Deep‐DIA. (C‐D) Gene Set Enrichment Analysis (GSEA) analysis of pathways enriched in ferroptosis (C) and autophagy (D) in Cas9+ FARAGE that express control or SH3GL1 sgRNAs by Deep‐DIA. (E) Kyoto Encyclopedia of Genes and Genomes (KEGG) enrichment scatter plot analysis of pathways enriched in ferroptosis in Cas9+ FARAGE that express control or SH3GL1 sgRNAs by Liquid chromatography–mass spectrometry (LC–MS). (F) Quantitative reverse transcription polymerase chain reaction (RT‐qPCR) analysis of TFRC, NCOA4, ferritin heavy chain 1 (FTH1) and GPX4 expression in FARAGE expressing control or SH3GL1 sgRNA. (G) Immunoblot analysis of TFRC, GPX4 and FTH1 expression in FARAGE and SUDHL8 expressing control or SH3GL1 sgRNA. (H) Immunoblot analysis of TFRC, GPX4 and FTH1 expression in FARAGE and SUDHL8 with control GFP and SH3GL1 cDNA. (I) Immunohistochemical (IHC) analysis of GPX4, NCOA4, FTH1 and TFRC expression in the subcutaneous tumour of FARAGE and SUDHL8 xenograft mouse model which express control or SH3GL1 sgRNAs. Brown signal in IHC was considered as positive staining. Data are shown as the mean ± SD. * *p* < .05; ** *p *< .01; *** *p* < .001; **** *p* < .0001 using one‐way analysis of variance (ANOVA) with multiple comparisons.

We have shown that SH3GL1 played vital roles in the survival of DLBCL cells and patients, however, the molecular mechanism remains unclear. To identify signalling pathways hijacked by SH3GL1 within DLBCL, Deep Data‐independent acquisition (Deep‐DIA) and liquid chromatography–mass spectrometry (LC–MS) were used on FARAGE cells with and without depleting of SH3GL1. Compared with control group, knocking out of SH3GL1 in FARAGE cell significantly upregulated 421 proteins and downregulated 72 proteins (Figure 3A). As illustrated in Figure 3A,B, which depict up and downregulated proteins, FTH1 was identified as one of the significantly downregulated proteins. GSEA enrichment analysis confirmed that ferroptosis and autophagy pathways were significantly enriched and activated in FARAGE cell with knocking out of SH3GL1 (Figure 3C,D). Kyoto Encyclopedia of Genes and Genomes (KEGG) pathway enrichment analysis of untargeted metabolomics also showed significantly enrichment of ferroptosis and glutathione metabolites after SH3GL1 deficiency in FARAGE cells (Figure 3E). These sequencing results implied that the depleting of SH3GL1 may lead to activated ferroptosis and autophagy pathways.

We next performed quantitative reverse transcription polymerase chain reaction (RT‐qPCR) to examine mRNA levels of the classically known ferroptosis‐related genes, including TFRC, NCOA4, FTH1 and GPX4 after depletion of SH3GL1 in FARAGE cells (Figure 3F). The results of RT‐qPCR showed that knocking out of SH3GL1 led to increased expression of TFRC and NCOA4, and decreased levels of FTH1 and GPX4. Like results of RT‐qPCR, SH3GL1 knockout also led to lower GPX4 and FTH1 and higher TFRC in protein levels (Figure 3G). Conversely, SH3GL1 overexpression resulted in decreased TFRC levels but increased GPX4 and FTH1 levels (Figure 3H). Subsequent IHC analysis of GPX4, FTH1 and TFRC expression in subcutaneous tumours from DLBCL xenograft model yielded similarly results (Figure 3I).

Overall, these results demonstrated that SH3GL1 is closely associated with ferroptosis and autophagy signalling pathways in DLBCL.

### 3.4 Knockout of SH3GL1 increases ferroptosis in DLBCL cells

**FIGURE 4:**
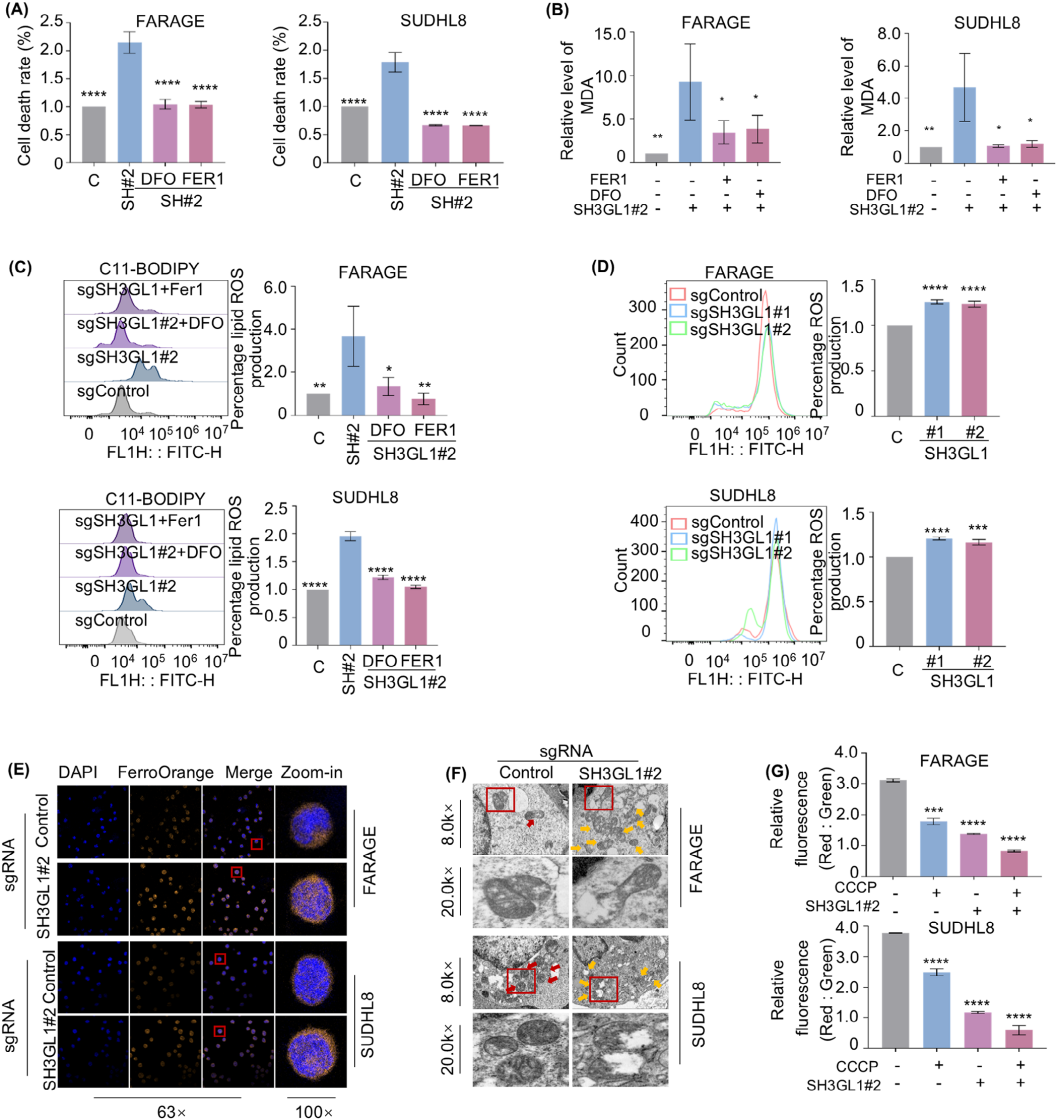
Knockout of SH3GL1 increases ferroptosis in diffuse large B‐cell lymphoma (DLBCL) cells. (A–C) FARAGE and SUDHL8 that express control or sgSH3GL1 were treated with DFO (3 µmol/L) and FER1 (1 µmol/L) for 48 h. Cell death (A) was assessed by PI stating followed by flow cytometry. Whole cell lysate was obtained to detect MDA levels (B) by microplate reader (OD 532 nm). Cells were stained with BODIPY 581/591 C11 and lipid reactive oxygen species (ROS) production (C) was assessed by flow cytometry. (D, E) Flow cytometry analysis of cellular ROS levels (D) and immunofluorescence analysis of cellular ferrous iron (E) in FARAGE and SUHDL8 expressing control or sgSH3GL1. (F) Transmission electron microscopy (TEM) analysis of mitochondria in subcutaneous tumour of a xenograft mouse model which was constructed by using FARAGE and SUDHL8 cells following control or SH3GL1 sgRNAs expression. Red arrow: normal mitochondria. Yellow arrow: damaged mitochondria (mitochondria with swelling, broken cristae and vacuolation). (G) Mitochondrial membrane potential (MMP) analysis in FARAGE and SUDHL8 expressing control or SH3GL1 sgRNAs without or with 1 µmol/L CCCP for 24 h by JC‐1. Data are shown as the mean ± SD. * *p* < .05; ** *p* < .01; *** *p* < .001; **** *p* < .0001 using one‐way analysis of variance (ANOVA) with multiple comparisons.

We next asked whether SH3GL1 depletion‐induced cell death is mediated through ferroptosis. FARAGE and SUDHL8 cells were treated with ferroptosis inhibitor (FER1) and deferoxamine (DFO) with and without knocking out of SH3GL1. Intriguingly, inhibition of ferroptosis completely rescued cell death caused by SH3GL1 depletion (Figure 4A). The accumulation of ROS, particularly lipid ROS is a typical feature of ferroptosis. We examined whether SH3GL1 expression was associated with ROS production. Malondialdehyde (MDA), the cellular terminal product of lipid peroxidation was obviously increased in sgSH3GL1#2 transfected FARAGE and SUDHL8 cells compared to controls. This effect was found to be reversible in the presence of FER1 or DFO (Figures 4B and S2A–C). Consistently, an increased accumulation of lipid ROS was observed in FARAGE and SUDHL8 cells with SH3GL1 knockout compared to controls, as evidenced by C11‐BODIPY staining. It also can be reversed in the presence of FER1 or DFO (Figures 4C and S2A–C). Flow cytometry analysis of DCF‐DA staining also demonstrated that SH3GL1 depletion significantly increased ROS production in DLBCL cells (Figures 4D and S2A–C).

Given that ferroptosis is specifically induced by ferrous iron, cellular ferrous iron levels were evaluated through immunofluorescence using FerroOrange probes. As illustrated in Figure 4E, FARAGE and SUDHL8 expressing sgSH3GL1 exhibited bright orange fluorescence in comparison to the control cells. This outcome implied that knockout of SH3GL1 may lead to an elevation intracellular ferrous iron in DLBCL cells. Given that alterations in mitochondrial structure are significant indicators of ferroptosis, transmission electron (TEM) was used to observe mitochondrial morphology in the subcutaneous tumours of the FARAGE and SUDHL8 xenograft mouse model (Figure 4F). The analysis revealed that the mitochondria did not reveal obvious morphological changes in control group (red arrows), whereas those in the SH3GL1 knockout group appeared swollen with disrupted cristae (yellow arrows). Flow cytometry analysis was conducted to assess the mitochondrial membrane potential (MMP) of DLBCL cells expressing control or SH3GL1 sgRNAs, both untreated and treated with CCCP, as an indicator of mitochondrial ATP generation. The results showed a significant decrease in MMP in FARAGE and SUDHL8 cells following SH3GL1 knockout (Figures 4G and S2A–2C).

These results suggested that knockout of SH3GL1 increased ferroptosis that leads to DLBCL cell death.

### 3.5 FTH1 is a key determinant for the loss of SH3GL1‐induced ferroptosis

Next, we endeavoured to elucidate the mechanism by which SH3GL1 inhibits ferroptosis. Ferroptosis is currently considered an autophagy‐dependent cell death.^28^, ^29^ Our proteomics results showed that FTH1, a pivotal protein involved in ferroptosis,^30^ was significantly affected upon depletion of SH3GL1. In addition, knocking out of SH3GL1 reduced FTH1 protein level, while overexpressing SH3GL1 increased FTH1 protein level as evidenced by Western blots (Figures 3G,H and S3A–C). Meanwhile, lower level of FTH1 was also observed in SH3GL1 knockout xenograft mouse tumour tissues (Figure 3I). Altogether our findings suggest that SH3GL1 modulates FTH1 expression in DLBCL cell lines.

**FIGURE 5:**
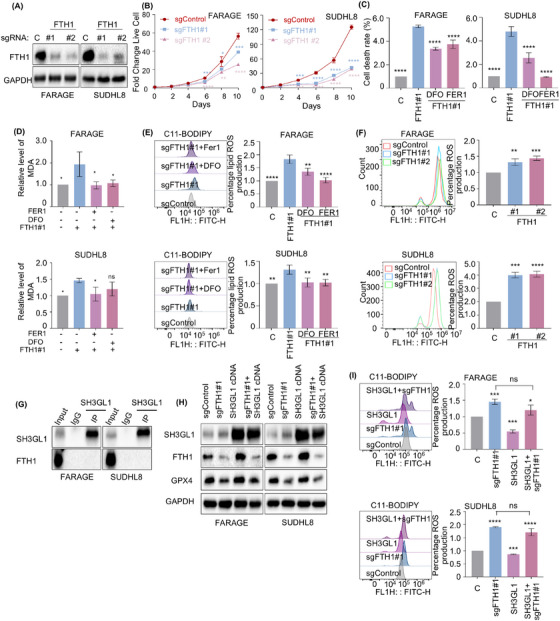
Ferritin heavy chain 1 (FTH1) is a key determinant for the loss of SH3GL1‐induced ferroptosis. (A) Immunoblot analysis showed the efficacy of FTH1 knockout in Cas9+ FARAGE and Cas9+ SUDHL8 expressing non‐targeting control (C) or sgRNA targeting FTH1 expression. (B) Growth curve analysis of FARAGE and SUDHL8 expressing control or FTH1 sgRNAs was performed at day 5 post‐sgRNA expression. (C–F) FARAGE and SUDHL8 that express control or sgFTH1 were treated with DFO (3 µmol/L) and FER1 (1 µmol/L) for 48 h. Cell death (C) was assessed by PI stating followed by flow cytometry. Whole cell lysate was obtained to detect MDA levels (D) by microplate reader (OD 532 nm). Cells were stained with BODIPY 581/591 C11 and lipid reactive oxygen species (ROS) production (E) was assessed by flow cytometry. (F) Flow cytometry analysis of cellular ROS levels in FARAGE and SUHDL8 expressing control or sgFTH1. (G) Coimmunoprecipitation analysis of SH3GL1 and FTH1 in FARAGE and SUDHL8. (H) Immunoblot analysis of GPX4 expression in FARAGE and SUDHL8 cells with SH3GL1 overexpression following FTH1 knockout. (I, J) Flow cytometry analysis of lipid ROS and cellular ROS production in FARAGE and SUDHL8 cells with SH3GL1 overexpression following FTH1 knockout. Data are shown as the mean ± SD. * *p* < .05; ** *p* < .01; *** *p* < .001; **** *p *< .0001 using one‐way analysis of variance (ANOVA) with multiple comparisons.

To further evaluate whether SH3GL1 regulates ferroptosis through FTH1, the role of FTH1 in ferroptosis in DLBCL was first examined. SgRNA targeting FTH1 efficiently depleted FTH1 from DLBCL cell lines (Figures 5A and S3A–C). Like the depletion of SH3GL1, knocking out of FTH1 also significantly impaired DLBCL cell outgrowth (Figures 5B and S3A–C). However, inhibition of ferroptosis rescued cell death by FTH1 depletion (Figures 5C and S3A–C). We also evaluated the role of FTH1 in MDA and lipid ROS. As expected, depletion of FTH1 increased the accumulation of MDA and lipid ROS in FARAGE and SUDHL8 cells, and this this effect were reversed in the presence of FER1 or DFO (Figures 5D,E and S3A–C). Flow cytometry analysis of DCF‐DA staining further confirmed that FTH1 knockout significantly increased ROS production in DLCBL cells (Figures 5F and S3A–C).

Subsequently, coimmunoprecipitation (CoIP) assay was used to further examine whether SH3GL1 directly interact with FTH1 in DLBCL cells, however, no direct binding was detected between SH3GL1 and FTH1 (Figure 5G). To further validated that SH3GL1 could inhibit DLBCL ferroptosis through FTH1, we overexpressed SH3GL1 following FTH1 knockout in DLBCL cells. The suppression of GPX4 expression by FTH1 was not reversed following SH3GL1 overexpression (Figure 5H). The flow cytometry analysis showed that the increase in lipid ROS production observed in FTH1 knockout cells was not eliminated following SH3GL1 overexpression (Figure 5I).

The knockout of FTH1, a crucial subunit of ferritin involved in iron storage, similarly increased sensitivity to ferroptosis in DLBCL cells, akin to the effects observed with SH3GL1 knockout. And SH3GL1 overexpression could not inhibit ferroptosis in FTH1 knockout DLBCL cells. This indicates a potential role of SH3GL1 in DLBCL cells ferroptosis through the modulation of FTH1‐mediated iron homeostasis, potentially contributing to the overload of cellular ferrous iron.

### 3.6 Knockout of SH3GL1 leads to ferritinophagy in DLBCL cells

**FIGURE 6:**
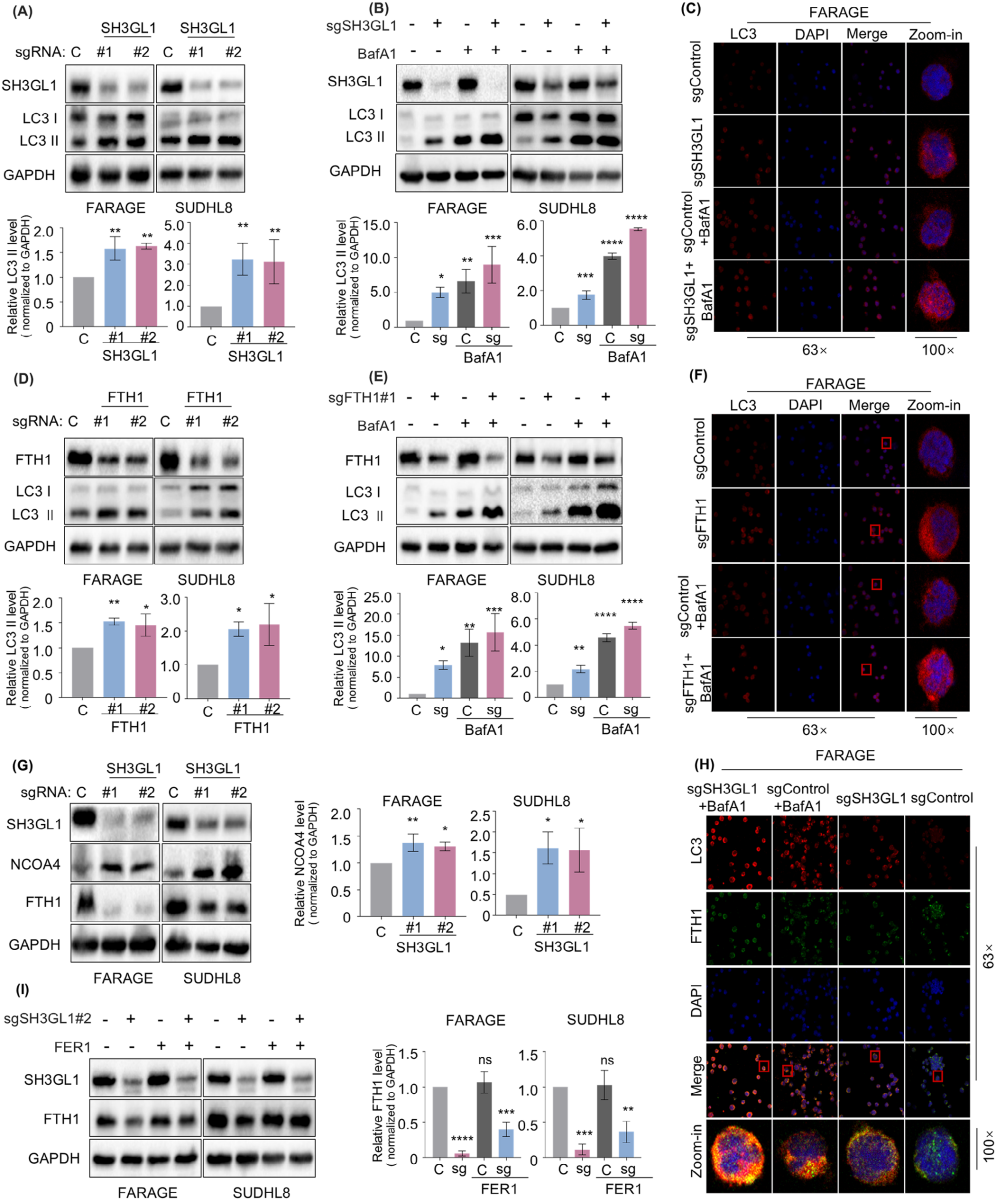
Knockout of SH3GL1 leads to ferritinophagy in diffuse large B‐cell lymphoma (DLBCL) cells (A) Immunoblot analysis of LC3 I/II expression in FARAGE and SUDHL8 that express control or SH3GL1 sgRNAs. (B) Immunoblot analysis of LC3 I/II expression in FARAGE and SUDHL8 that express control or SH3GL1 sgRNAs with or without Bafilomycin A1 (BafA1) (100 nm) for 24 h. (C) Immunofluorescence analysis of LC3 expression in FARAGE that express control or SH3GL1 sgRNA with or without BafA1 (100 nm) for 24 h. (D) Immunoblot analysis of LC3 I/II expression in FARAGE and SUDHL8 that express control or ferritin heavy chain 1 (FTH1) sgRNAs. (E) Immunoblot analysis of LC3 I/II expression in FARAGE and SUDHL8 that express control or FTH1 sgRNAs with or without BafA1 (100 nm) for 24 h. (F) Immunofluorescence analysis of LC3 expression in FARAGE that express control or FTH1 sgRNA with or without BafA1 (100 nm) for 24 h. (G) Immunoblot analysis of NCOA4 and FTH1 expression in FARAGE and SUDHL8 that express control or SH3GL1 sgRNAs. (H) Immunofluorescence analysis of LC3 and FTH1 expression in FARAGE that express control or SH3GL1 sgRNA with or without BafA1 (100 nm) for 24 h. (I) Immunoblot analysis of FTH1 expression in FARAGE and SUDHL8 that express control or SH3GL1 sgRNAs with or without FER1 (1 µm) for 48 h. Data are shown as the mean ± SD. * *p* <.05; ** *p* <.01; *** *p* <.001; **** *p* <.0001 using one‐way analysis of variance (ANOVA) with multiple comparisons.

Ferritinophagy is a form of selective autophagy that facilitates the degradation of intracellular ferritin. The process is primarily mediated by FTH1 and the cargo receptor NCOA4. The GSEA analysis from proteomics and KEGG analysis from metabolomics showed that depleting of SH3GL1 activate ferroptosis and autophagy pathways. Therefore, we speculated that SH3GL1 knockout causes ferritinophagy in DLBCL cells. To test this, we first investigated whether SH3GL1 plays a role in cell autophagy. The autophagosome marker LC3‐II was upregulated in SH3GL1 knockout groups compared to control group in SUDHL8 and FARAGE cell lines (Figure 6A). To further substantiate this finding, cells were exposed to 100 nM Bafilomycin A1 (BafA1) for 24 h to inhibit the degradation of autophagosome. Then expression level of LC3‐II was examined to quantify the autophagosome levels. Western blot analysis demonstrated that depletion of SH3GL1 significantly increased LC3‐II levels in the presence of BafA1, suggesting that silencing of SH3GL1 enhances autophagy during early developmental stages (Figure 6B). The immunofluorescence analysis showed similarly results as Western blot analysis in FARAGE (Figure 6C).

Since knockout of SH3GL1 decreased the expression of FTH1, leading to degradation of ferritin, our study proceeded to investigate the effects of FTH1 knockout on autophagy in DLBCL cells. The analysis of Western blot analysis revealed that FTH1 knockout led to an increased in LC3‐II expression, and BafA1 pretreatment further increased the expression of LC3‐II in DLBCL cells expressing sgFTH1 (Figure 6D,E). Immunofluorescence analysis corroborated these findings (Figure 6F). These results implied that silencing of SH3GL1 may promote autophagy through FTH1.

NCOA4 acts as a selective cargo receptor directing ferritin to the lysosome for iron release and autophagy‐mediated degradation (referred to as ferritinophagy).^20^ Depletion of SH3GL1 increased NCOA4 protein level, indicating that the silencing of SH3GL1 promotes the activation of ferritinophagy (Figure 6G). Immunofluorescence analysis revealed that the knockout of SH3GL1 induced an enhanced localisation of LC3 and a reduced localisation of FTH1. Furthermore, treatment with BafA1 resulted in an increased in the colocalisation of FTH1 and LC3 (Figure 6H). The results of Western blot also showed that the decrease in FTH1 expression was reversed by the addition of FER1 in FARAGE and SUDHL8 cells expressing SH3GL1 sgRNA (Figure 6I).

These results collectively indicated that SH3GL1 plays a role in inducing autophagic‐dependent degradation of FTH1, which was essential for the SH3GL1‐induced ferroptotic death of DLBCL cells.

### 3.7 SH3GL1 overexpression inhibited doxorubicin‐induced ferroptosis in DLBCL cells

Although DLBCL is curable, 40%–50% of the patients suffer from relapsed/refractory disease and have poor survivals. Chemotherapy resistance is the major reason and detailed mechanisms have not been well defined. Previous study reported that cancer cells that develop resistance have been shown to have increased sensitivity to ferroptosis.^13^ Doxorubicin is an important chemotherapeutic agent for treating DLBCL. Subsequently, examinations were used to explore the association between SH3GL1 and doxorubicin resistance.

**FIGURE 7:**
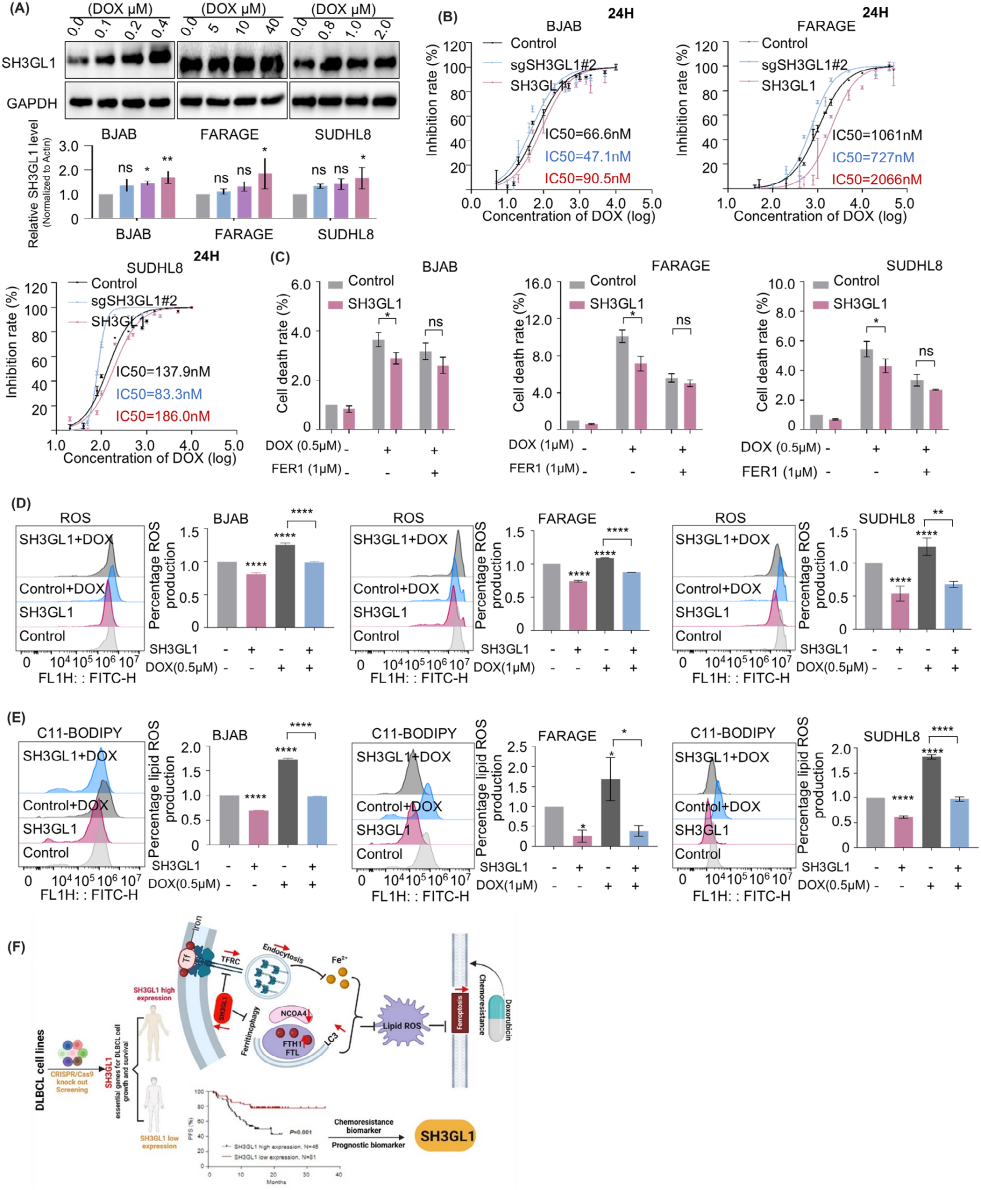
SH3GL1 overexpression inhibited doxorubicin‐induced ferroptosis in diffuse large B‐cell lymphoma (DLBCL) cells. (A) Immunoblot analysis of SH3GL1 expression after treatment with different concentration of doxorubicin in BJAB, FARAGE and SUDHL8. (B) Inhibitory rates of BJAB, FARAGE and SUDHL8 expressing SH3GL1 sgRNA or SH3GL1 cDNA was analysed by CCK8 assay 24 h after treatment with different concentration of doxorubicin (OD 450 nm). (C) Flow cytometry analysis of cells death rate was measured using PI staining in BJAB, FARAGE and SUDHL8 with or without SH3GL1 overexpression after treatment with doxorubicin (500 nm or 1 µm) plus either DMSO or FER1 (1 µm). (D, E) Flow cytometry analysis of lipid reactive oxygen species (ROS) production (D) and cellular ROS levels (E) in BJAB, FARAGE and SUDHL8 with or without SH3GL1 overexpression after treatment with doxorubicin (500 nm or 1 µm). (F) Schematic model of SH3GL1‐activated ferritin heavy chain 1 (FTH1) inhibits ferroptosis and confers doxorubicin resistance in DLBCL. Data are shown as the mean ± SD. * *p* <.05; ** *p* <.01; *** *p* <.001; **** *p* <.0001 using one‐way analysis of variance (ANOVA) with multiple comparisons.

Elevated protein expression levels of SH3GL1 were identified in BJAB, FARAGE and SUHDL8 cell lines following treatment with varying concentrations of doxorubicin (Figure 7A). Cell counting Kit‐8 (CCK‐8) assay was utilised to test the effect of SH3GL1 on DLBCL cells resistance to doxorubicin. The finding showed that the IC50 values of doxorubicin was significantly elevated in DLBCL cells overexpressing SH3GL1 and reduced in cells depleted with SH3GL1 (Figure 7B). These results suggested that doxorubicin treatment facilitate the upregulation of SH3GL1, and high expression of SH3GL1 inhibits doxorubicin‐induced death in DLBCL cells.

Previous studies have identified a correlation between doxorubicin and ferroptosis in DLBCL cells,^31^ however, the underlying antitumour mechanisms by which doxorubicin induces ferroptosis remain to be elucidated. Here, using PI staining, we demonstrated that the doxorubicin‐induced cell death was attenuated by SH3GL1 overexpression. Additionally, the addition of FER1 to SH3GL1 overexpressing cells treated with doxorubicin further attenuated the doxorubicin‐induced cell death (Figure 7C). Our findings also showed that doxorubicin treatment resulted in increased levels of cellular ROS and lipid ROS, suggesting that doxorubicin could induce ferroptosis in DLBCL cells. Simultaneously, the overexpression of SH3GL1 mitigated the ferroptosis induced by doxorubicin treatment (Figure 7D,E).

Altogether, these results revealed that doxorubicin facilitated the upregulation of SH3GL1, and the overexpression of SH3GL1 inhibited doxorubicin‐induced ferroptosis, thereby reducing the sensitivity of DLBCL cells to doxorubicin.

## 4 DISCUSSION

This research identified that high expression of SH3GL1 was associated with poor prognosis of patients with DLBCL. Functional experiments confirmed the significant role of SH3GL1 in progression of DLBCL. Further investigations demonstrated the relevance of SH3GL1 in the regulation of ferroptosis, particularly FTH1‐mediated ferroptosis in DLBCL. Moreover, the overexpression of SH3GL1 was found to inhibit doxorubicin‐induced ferroptosis in DLBCL. Therefore, this study highlights SH3GL1 as a novel regulator of ferroptosis in DLBCL, suggesting that targeting SH3GL1 may be a promising strategy to prevent DLCBL progression and overcome the resistance to traditional cancer treatments (Figure 7F).

By delving into the DepMap database, we found that SH3GL1 is an essential gene in over ten DLBCL cell lines. Deficiency of SH3GL1 can impair in vitro B‐cell proliferation, and the production of antibody in human.^32^ SH3GL1 is involved in the tumourigenesis of cancers, such as osteosarcoma^33^ and breast cancer.^7^, ^8^ However, the role of SH3GL1 in the pathogenesis of DLBCL remains unclear. In this current study, our findings showed that SH3GL1 was upregulated in lymphoma cell lines, including DLBCL cell lines, CLL cell lines, MCL cell lines, BL cell lines and ALCL cell lines. To study the function of SH3GL1, CRISPR/Cas9 editing was performed to knock out SH3GL1 in multiple DLBCL cell lines, including BJAB, FARAGE, KIS‐1, SUDHL4 and SUDHL8. Our results demonstrated that the knockout of SH3GL1 significantly inhibited DLBCL cell proliferation and induced cell death, as evidenced by both in vivo and in vitro assays. However, depletion of SH3GL1 in MEC‐1 and KARPARS299, a CLL and ALCL cell line, did not affect their proliferation, suggesting the specificity of SH3GL1 for DLBCL survivals. In the clinic, IHC was used to analyse 126 paraffin‐embedded samples from patients with DLBCL. The results indicated that elevated expression of SH3GL1 was significantly associated with poor PFS and OS in DLBCL patients. And patients with elevated levels of SH3GL1 expression were more predisposed to developing resistance to chemotherapy and progressing to progressive disease. Ferroptosis is a recently identified form of cell death distinguished by its reliance on iron‐dependent lipid peroxidation.

Ferroptosis is a recently identified form of cell death distinguished by its reliance on iron‐dependent lipid peroxidation. It is well established that ferroptosis plays a crucial role in biological processes of cancers, including DLBCL and peripheral T cell lymphoma.^34^, ^35^, ^36^, ^37^, ^38^, ^39^ Canonical ferroptosis induction involves the inactivation of primary protective mechanism that safeguard cellular membranes against peroxidative damage, while the non‐canonical pathway induces ferroptosis through the augmentation of labile iron pool.^40^ Consequently, increased iron levels can enhance the susceptibility to ferroptosis. Numerous genes or proteins involved in iron haemostasis, encompassing its import, export and storage, have been shown to influence sensitivity to ferroptosis. In the present study, our findings revealed for the first time that the knockout of SH3GL1 resulted in ferroptosis in DLBCL cells, with the underlying mechanism involving the modulation of FTH1‐mediated iron homeostasis. The principal intracellular iron‐storage protein, FTH1, is crucial for maintaining cellular iron homeostasis and is implicated in the prognosis of various types of cancer, including pancreatic cancer, lung cancer, liver cancer and others.^41^, ^42^, ^43^ Our research demonstrated that knockout of SH3GL1 leads to decreased expression of FTH1, leading to elevated intracellular iron levels, causing ferroptosis.

Ferritinophagy is a selective autophagic process that degrades intracellular ferritin, leading to increased cellular iron levels, causing ferroptosis. Ferritinophagy is mainly controlled by FTH1 and the cargo receptor NCOA4. Many studies have highlighted the critical role of ferritinophagy in maintaining intracellular iron homeostasis, as well as its significant association with the occurrence and development of cancers.^44^, ^45^, ^46^ Our finding showed that SH3GL1 inhibits NCOA4 expression which in turn abolished ferritinophagy‐induced ferroptosis.

Although the standard first‐line R‐CHOP regimen has shown efficiency in treating approximately 60% of DLBCL patients, the remaining patients suffer from relapsed/refractory disease and poor survival outcomes due to resistance to this regimen. Doxorubicin plays a fundamental role in the R‐CHOP regimen. We examined the expression level of SH3GL1 in DLBCL tissues exhibiting varying responses to chemotherapy, as presented in Figure 2. The findings underscore a significant correlation between high SH3GL1 expression and chemotherapy resistance, especially doxorubicin resistance.

Previous studies have identified a strong correlation between ferroptosis and doxorubicin, suggesting ferroptosis may help alleviate doxorubicin resistance in various cancers, including breast cancer, osteosarcoma and DLBCL.^31^, ^47^, ^48^, ^49^ Our findings demonstrated that doxorubicin treatment increased SH3GL1 expression in DLBCL cells, indicating that SH3GL1 might involve in the modulation of doxorubicin‐induced cell death. Indeed, our results revealed that doxorubicin treatment can trigger ferroptosis, and SH3GL1 overexpression mitigated the effects of doxorubicin‐induced ferroptosis in DLBCL cell lines, thereby reducing the sensitivity of DLBCL cells to doxorubicin. These findings underscored the pivotal role of SH3GL1 in mediating chemotherapy‐induced ferroptosis.

In this study, we uncovered a correlation between elevated SH3GL1 expression in DLBCL patients and a negative prognosis. Our research presents convincing evidence that SH3GL1 is important in promoting DLBCL cell survival through the regulation of FTH1‐mediated ferroptosis. Furthermore, high expression of SH3GL1 was found to attenuate the effects of doxorubicin‐induced ferroptosis. Taken together, our results emphasise the significance of SH3GL1 as a valuable prognostic biomarker and a potential target for anti‐DLBCL intervention in the future.

## AUTHOR CONTRIBUTIONS

Zi‐Wen Duan, Wei‐Ting Wang, Yan Wang, Rong Wang, Wei Hua and Chun‐Yu Shang performed the experiments. Zi‐Wen Duan, Yan Wang, Wei Hua and Rui Gao provided technological assistance. Zi‐Wen Duan, Hao‐Rui Shen, Yue Li, Jia‐Zhu Wu, Hua Yin, Li Wang and Jian‐Yong Li collected the clinical data for patients. Zi‐Wen Duan and Yan Wang prepared the manuscript. Jin‐Hua Liang and Wei Xu supervised the study. All the authors reviewed and approved the manuscript.

## CONFLICT OF INTEREST STATEMENT

The authors declare no conflicts of interest.

## ETHICS STATEMENT

All animal experiments were conducted in compliance with the guidelines set forth by the Institutional Animal Care and Use Committee of Nanjing Medical University (Ethics Approval Number: IACUC‐2310063), adhering to the regulations established by the National Institutes of Health (NIH). Primary tumour samples from 126 patients with DLBCL were obtained from the First Affiliated Hospital of Nanjing Medical University with informed consent and approval from the Ethics Committee of the Institutional Review Broad of Jiangsu Province Hospital (No. 2024‐SRFA‐3‐8).

## Supporting information



Supporting Information

Supporting Information

Supporting Information

Supporting Information

Supporting Information

Supporting Information

Supporting Information

Supporting Information

## Data Availability

The datasets analysed during the current study are available from the corresponding author upon reasonable request.
