# Supplementary figures and images for: SH3GL1‐activated FTH1 inhibits ferroptosis and confers doxorubicin resistance in diffuse large B‐cell lymphoma
Source: Clin Transl Med. 2025 Mar 4;15(3):e70246. doi: 10.1002/ctm2.70246 (PMC11879899; doi:10.1002/ctm2.70246)

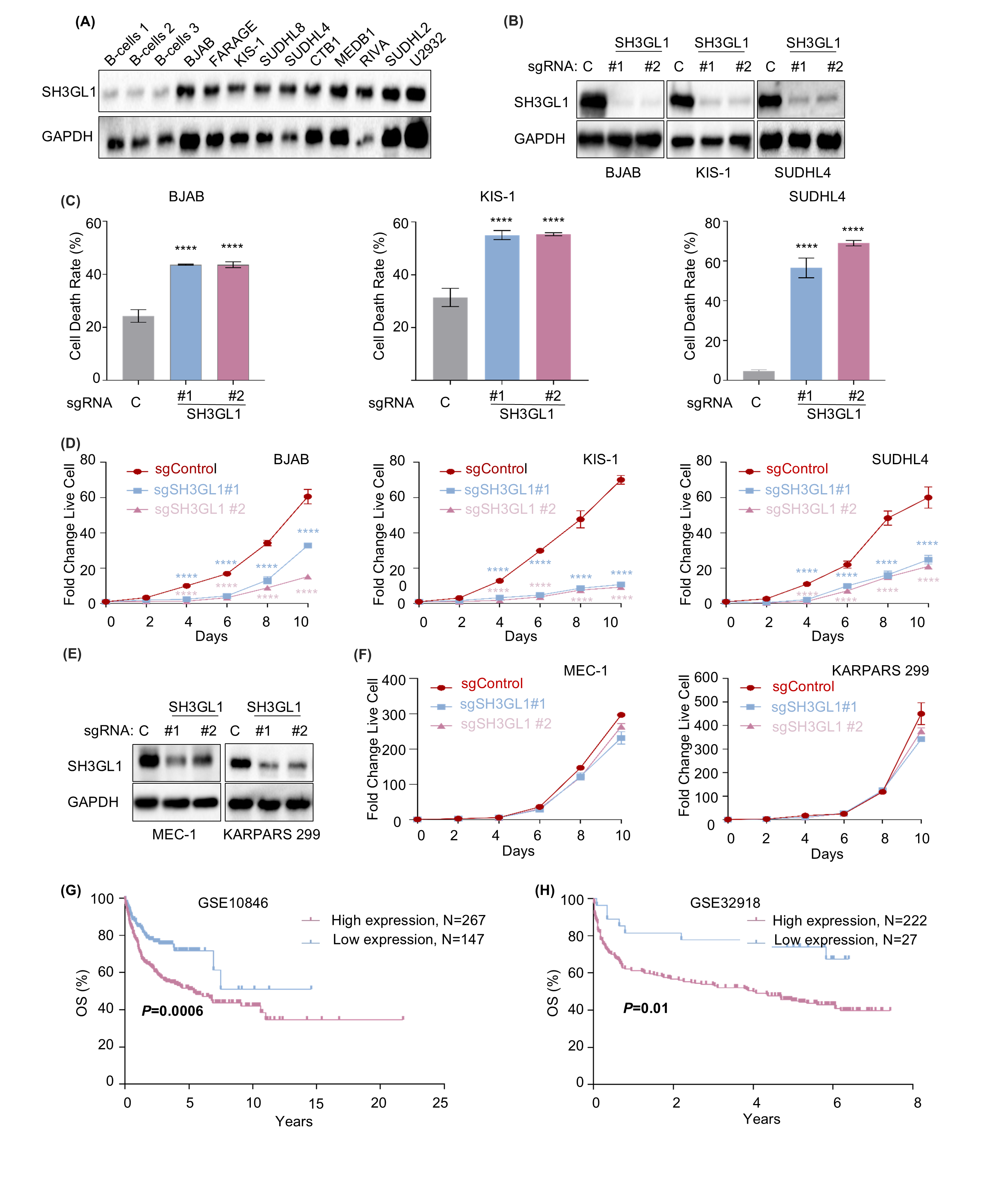

Supplement: Supplementary file 1 — Supporting Information [file CTM2-15-e70246-s006.TIF]

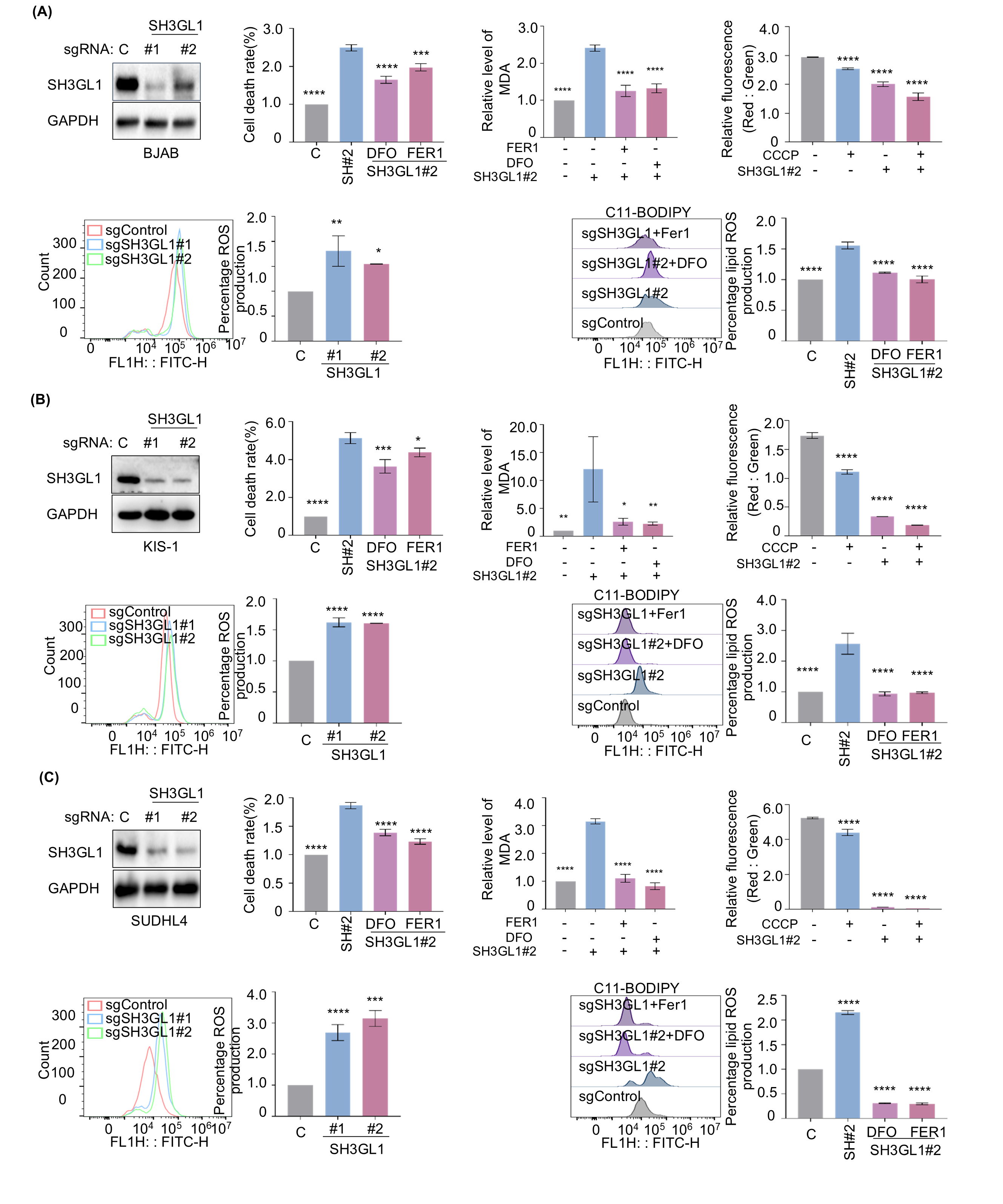

Supplement: Supplementary file 2 — Supporting Information [file CTM2-15-e70246-s003.TIF]

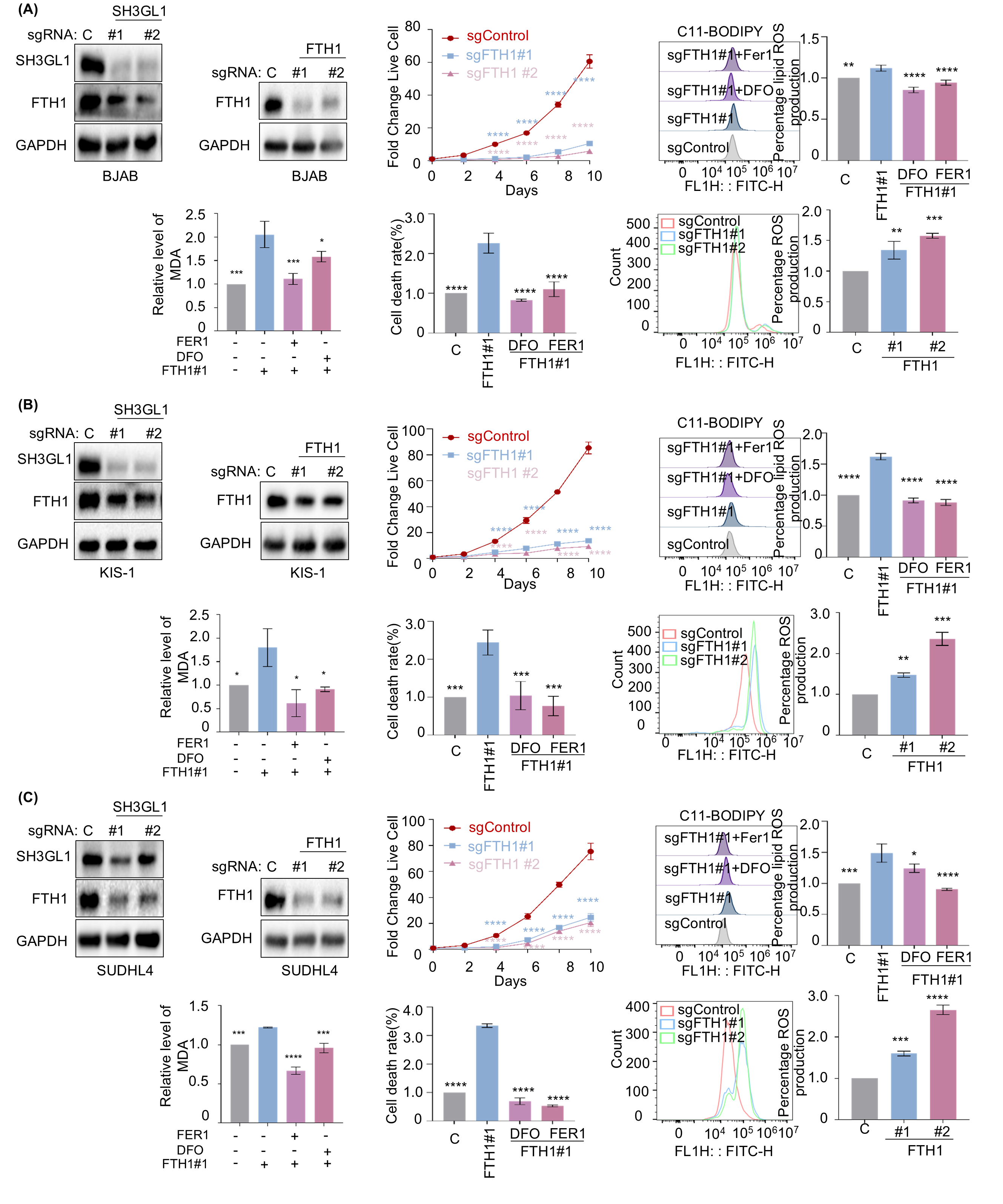

Supplement: Supplementary file 3 — Supporting Information [file CTM2-15-e70246-s002.TIF]
