# Supplementary material for: SH3GL1‐activated FTH1 inhibits ferroptosis and confers doxorubicin resistance in diffuse large B‐cell lymphoma
Source: Clin Transl Med. 2025 Mar 4;15(3):e70246. doi: 10.1002/ctm2.70246 (PMC11879899; doi:10.1002/ctm2.70246)
Supplement: Supplementary file 4 — Supporting Information [file CTM2-15-e70246-s007.docx]

**Supplemental materials and methods**

**RNA-sequencing and Liquid chromatography-mass spectrometry (LC-MS)**

RNA sequencing was carried out by Novogene using the Illumina NovaSeq 6000 platform, as detailed in prior reports. A total of six samples were utilized for RNA-sequencing in this study, with three samples from the sgControl group and three from the sgSH3GL1 group [1].

LC-MS analysis was conducted by Novogene and processed using the LC-MS/MS system according to established protocols [1]. Additionally, a total of twelve samples were employed for LC-MS analysis, with six samples from each of the sgControl and six from the sgSH3GL1 groups.

**CRISPR/Cas9 gene editing**

SgRNAs were cloned into the lentiGuide-PURO vector (Addgene, 52963). Prior to CRISPR/Cas9 gene editing, Cas9 stable cell lines were established through the transduction of cells with lentiviruses produced by lentiCas9-Blast (Addgene, 52962). Lentiviruses expressing sgRNA were generated via HEK293T transfection. The transfection medium was replaced with RPMI-1640 containing 10% fetal bovine serum 24 hours post-transfection. Lentiviruses were collected at 48- and 72-hours post-transfection. Prior to transducing cell lines with stable Cas9 expression, the virus supernatant was filtered using a 0.45 μm centrifugal filter device (Millipore, RINB72613). Puromycin-resistant cells were selected using puromycin (InvivoGen, ant-pr-1) at a concentration of 3mg/ml for a period of 5‒7 days. The loss of protein expression of the target gene was confirmed through western blot analysis.

**cDNA overexpression**

The SH3GL1 cDNA in the entry vector and control vector pCDH-PURO-GFP were sourced from Youbio. The recombinant plasmid containing the full-length human SH3GL1 cDNA was cloned into the pCDH-PURO vector obtained from Youbio. Lentiviruses were produced by transfecting HEK293T cells with either pCDH-PURO-GFP or -SH3GL1, and subsequently used for cell transduction. Selection of transduced cells was carried out using puromycin (InvivoGen, ant-pr-5b) for a duration of two weeks. Overexpression of SH3GL1 was confirmed by western blotting.

**Quantitative RT-PCR**

Total RNA was extracted from collected cells using the RNA Isolation Total RNA Extraction Reagent following previously established protocols [2]. Primer sequences were provided in a Table S4 for reference, with ACTIN serving as the internal control gene.

**Transmission electron microscopy (TEM)**

Fresh tissues were prepared as outlined in prior studies [2] and examined using TEM (HITACHI, HT7800/HT7700).

**Coimmunoprecipitation assay (CoIP)**

CoIP assay was conducted in accordance with the manufacturer's instructions for the IP/CoIP Kit (Absin, abs955). Cell lysates were chilled on ice and incubated with SH3GL1 (Santa Cruz, sc-365704) or normal mouse IgG (CST, 61656S) overnight at 4°C with rotation, followed by the addition of agarose beads conjugated with protein A/G for 3- and 4- hours at 4°C with gentle rotation. Samples were analyzed by immunoblotting.

**Cell death assay**

The cells subjected to different treatments were harvested, washed twice with phosphate-buffered saline (PBS), and resuspended in binding buffer. Propidium iodide (PI) staining solution (Vazyme, A214-02) was then added and the cells were incubated at room temperature for 15 minutes in the dark. Subsequently, 400 µl of binding buffer was added to each tube. Cell death assays were conducted using flow cytometry (Beckman Coulter, GALLIOS) within 1 hour, and the results were analyzed using Flowjo software V10.

**Reactive oxygen species (ROS) analysis**

The differently treated cells were collected and incubated with 10 μmol 2',7'-dichlorofluorescin diacetate (DCFH-DA) (Beyotime, S0033S) in serum-free RPMI-1640 medium at 37 °C for 30 minutes in the dark. Following three washes with serum-free RPMI1640 medium, intracellular ROS levels were assessed using flow cytometry (Beckman Coulter, GALLIOS). Data analysis was conducted using Flowjo software V10.

**Malondialdehyde (MDA) assay**

The MDA assay kit (Beyotime, S0131M) was utilized to measure lipid peroxidation levels as per the manufacturer's instructions. Briefly, cells treated with various conditions were harvested and incubated with 150 μL lysis buffer on ice for 15 minutes. Subsequently, 100 μl of sample supernatant was combined with 200 μl of TBA working solution and heated at 100 °C for 15 minutes, while the remaining supernatant was used for protein concentration determination. Following centrifugation, the supernatant was retrieved and the concentration of MDA was quantified by measuring the absorbance at 532 nm. The MDA concentration was determined and the relative MDA content was calculated as the ratio of MDA concentration to protein concentration.

**Lipid peroxidation measurement**

Cells subjected to various treatments were collected, incubated with 5 μmol BODIPY 581/591 C11 (Dojindo, L267) for 30 minutes, washed with PBS, and harvested by centrifugation. Lipid peroxidation levels were evaluated using flow cytometry (Beckman Coulter, GALLIOS), and data analysis was performed using Flowjo software V10.

**Cellular ferrous iron detection**

Cellular ferrous iron levels were quantified using FerroOrange probes (Dojindo, F374) according to manufacturer's instructions. Briefly, cells were seeded onto 12-well plates and subjected to the specified treatments. Subsequently, cells were collected and rinsed thrice with HBSS, exposed to 1 μmol/L FerroOrange for 30 minutes at 37 °C, 5% CO2 in a controlled environment, and promptly imaged using the LEICA Stellaris STED system followed by image analysis with the LAS X software.

**Analysis of mitochondrial membrane potential (MMP)**

MMP was quantified using a JC-1-based enhanced mitochondrial membrane potential assay kit (Beyotime, C2003S) according to the manufacturer's protocol. The MMP was determined by calculating the ratio of red fluorescence (JC-1 polymer) to green fluorescence (JC-1 monomer). Flow cytometry analysis was performed on the samples using a Beckman Coulter GALLIOS instrument, and the data were analyzed using Flowjo software version 10.

**References**

[1] J.H. Liang, Y.M. Ren, K.X. Du, R. Gao, Z.W. Duan, J.R. Guo, et al., MYC-induced cytidine metabolism regulates survival and drug resistance via cGas-STING pathway in mantle cell lymphoma, Br. J. Haematol. 202 (3) (2023) 550–565.

[2] W.T. Wang, J.R. Guo, L. Wang, J.Z. Wu, H.R. Shen, Y.L. Kong, et al., EBV-Mir-BART5-5p targets p53 independent pathway in cytoplasm: potential role in EBV lymphomagenesis, Genes Dis 10 (4) (2023) 1154–1156.

**Supplementary Figure Legends**

**Figure S1. SH3GL1 promotes DLBCL cell proliferation**

(A) Immunoblot analysis showed high expression of SH3GL1 protein in DLBCL cell lines and low expression in CD19+ B cells which were isolated from normal human peripheral blood mononuclear cells.

(B) Immunoblot analysis showed the efficacy of SH3GL1 knockout in Cas9+ BJAB, KIS-1 and SUDHL4 expressing non-targeting control (C) or sgRNA targeting SH3GL1 expression.

(C) Flow cytometry analysis of cell death was measured using PI Kit in BJAB, KIS-1 and SUDHL4 at Day 7 postexpression of control or sgRNAs targeting SH3GL1.

(D) Growth curve analysis of BJAB, KIS-1 and SUDHL4 expressing control or SH3GL1 sgRNAs was performed at day 5 post-sgRNA expression.

(E) Immunoblot analysis showed the efficacy of SH3GL1 knockout in Cas9+ MEC-1 and KARPARS 299 expressing non-targeting control (C) or sgRNA targeting SH3GL1 expression.

(F) Growth curve analysis of MEC-1 and KARPARS 299 expressing control or SH3GL1 sgRNAs was performed at day 5 post-sgRNA expression.

(G) Overall survival (OS) of DLBCL patients based on SH3GL1 expression GSE10846 and GSE32918 databases.

Data are shown as the mean ± SD. *, P < 0.05; **, P < 0.01; ***, P < 0.001; ****, P < 0.0001 using one way ANOVA with multiple comparisons.

**Figure S2. Knockout of SH3GL1 increases ferroptosis in DLBCL cells**

(A) Knockout of SH3GL1 can induce ferroptosis in BJAB cells. Cell death was assessed by PI stating followed by flow cytometry, whole cell lysate was obtained to detect MDA levels by microplate reader (OD 532 nm), cells were stained with BODIPY 581/591 C11 and lipid ROS production was assessed by flow cytometry, cells were stained with DCF-DA and cellular ROS levels was assessed by flow cytometry, and cells were stained with JC-1 and mitochondrial membrane potential was assessed by flow cytometry.

(B) Knockout of SH3GL1 can induce ferroptosis in KIS-1 cells. Cell death was assessed by PI stating followed by flow cytometry, whole cell lysate was obtained to detect MDA levels by microplate reader (OD 532 nm), cells were stained with BODIPY 581/591 C11 and lipid ROS production was assessed by flow cytometry, cells were stained with DCF-DA and cellular ROS levels was assessed by flow cytometry, and cells were stained with JC-1 and mitochondrial membrane potential was assessed by flow cytometry.

(C) Knockout of SH3GL1 can induce ferroptosis in SUDHL4 cells. Cell death was assessed by PI stating followed by flow cytometry, whole cell lysate was obtained to detect MDA levels by microplate reader (OD 532 nm), cells were stained with BODIPY 581/591 C11 and lipid ROS production was assessed by flow cytometry, cells were stained with DCF-DA and cellular ROS levels was assessed by flow cytometry, and cells were stained with JC-1 and mitochondrial membrane potential was assessed by flow cytometry.

Data are shown as the mean ± SD. *, *P* < 0.05; **, *P* < 0.01; ***, *P* < 0.001; ****, P < 0.0001 using one way ANOVA with multiple comparisons.

**Figure S3. FTH1 is a key determinant for the loss of SH3GL1-induced ferroptosis**

(A) Knockout of SH3GL1 decreased FTH1 expression in BJAB cells, and knockout of FTH1 can induce ferroptosis in BJAB cell. Cell death was assessed by PI stating followed by flow cytometry and growth curve analysis, whole cell lysate was obtained to detect MDA levels by microplate reader (OD 532 nm), cells were stained with BODIPY 581/591 C11 and lipid ROS production was assessed by flow cytometry, cells were stained with DCF-DA and cellular ROS levels was assessed by flow cytometry, and cells were stained with JC-1 and mitochondrial membrane potential was assessed by flow cytometry.

(B) Knockout of SH3GL1 decreased FTH1 expression in KIS-1 cells, and knockout of FTH1 can induce ferroptosis in KIS-1 cell. Cell death was assessed by PI stating followed by flow cytometry and growth curve analysis, whole cell lysate was obtained to detect MDA levels by microplate reader (OD 532 nm), cells were stained with BODIPY 581/591 C11 and lipid ROS production was assessed by flow cytometry, cells were stained with DCF-DA and cellular ROS levels was assessed by flow cytometry, and cells were stained with JC-1 and mitochondrial membrane potential was assessed by flow cytometry.

(C) Knockout of SH3GL1 decreased FTH1 expression in SUDHL4 cells, and knockout of FTH1 can induce ferroptosis in SUDHL4 cell. Cell death was assessed by PI stating followed by flow cytometry and growth curve analysis, whole cell lysate was obtained to detect MDA levels by microplate reader (OD 532 nm), cells were stained with BODIPY 581/591 C11 and lipid ROS production was assessed by flow cytometry, cells were stained with DCF-DA and cellular ROS levels was assessed by flow cytometry, and cells were stained with JC-1 and mitochondrial membrane potential was assessed by flow cytometry.

Data are shown as the mean ± SD. *, *P* < 0.05; **, *P* < 0.01; ***, *P* < 0.001; ****, *P* < 0.0001 using one way ANOVA with multiple comparisons.
