# Supplementary material for: SH3GL1‐activated FTH1 inhibits ferroptosis and confers doxorubicin resistance in diffuse large B‐cell lymphoma
Source: Clin Transl Med. 2025 Mar 4;15(3):e70246. doi: 10.1002/ctm2.70246 (PMC11879899; doi:10.1002/ctm2.70246)
Supplement: Supplementary file 5 — Supporting Information [file CTM2-15-e70246-s008.docx]

| Table S1. List of antibodies | |  |  |  |
| --- | --- | --- | --- | --- |
| Gene | Company | Catalog# | Dilution | |
|  |  |  | WB | IHC |
| anti-SH3GL1 | Santa Cruz | sc-365704 | 1:250 | 1:250 |
| anti-FTH1 | Santa Cruz | sc-376594 | 1:100 | 1:400 |
| anti-NCOA4 | Cell signaling Technology | 66849S | 1:1000 | 1:50 |
| anti-GPX4 | Abcam | ab125066 | 1:1000 | 1:50 |
| anti-TFRC | Abcam | ab214039 | 1:2500 | 1:500 |
| anti-LC3 | Proteintech | 14600-1-AP | 1:2500 | - |
| anti-GAPDH | Proteintech | 60004-1-Ig | 1:10000 | - |
