# Supplementary material for: SH3GL1‐activated FTH1 inhibits ferroptosis and confers doxorubicin resistance in diffuse large B‐cell lymphoma
Source: Clin Transl Med. 2025 Mar 4;15(3):e70246. doi: 10.1002/ctm2.70246 (PMC11879899; doi:10.1002/ctm2.70246)
Supplement: Supplementary file 6 — Supporting Information [file CTM2-15-e70246-s001.docx]

| Table S2. List of reagents |  |  |
| --- | --- | --- |
| Reagents | Company | Catalog# |
| Lipid Peroxidation Probe-BDP 581/591 C11 | Dojindo | L267 |
| FerroOrange | Dojindo | F374 |
| Lipid Peroxidation MDA Assay Kit | Beyotime | S0131M |
| ROS Assay Kit | Beyotime | S0033S |
| JC-1 Assay Kit | Beyotime | C2003S |
| Deferoxamine mesylate | Selleck | S5742 |
| Doxorubicin hydrochloride | MedChemExpress | HY-15142 |
| Ferrostatin-1 | MedChemExpress | HY-100579 |
| CCCP | MedChemExpress | HY-100941 |
| Bafilomycin A1 | MedChemExpress | HY-100558 |
