# Supplementary material for: SH3GL1‐activated FTH1 inhibits ferroptosis and confers doxorubicin resistance in diffuse large B‐cell lymphoma
Source: Clin Transl Med. 2025 Mar 4;15(3):e70246. doi: 10.1002/ctm2.70246 (PMC11879899; doi:10.1002/ctm2.70246)
Supplement: Supplementary file 7 — Supporting Information [file CTM2-15-e70246-s004.docx]

| Table S3. Sequences of primers and sgRNAs used for CRISPR editing | | | |
| --- | --- | --- | --- |
|  | Name | sgRNA sequence | Source |
| sgRNA | sgControl SS | ATTTCGCAGATCATCGACAT | Brunello Library |
|  | SH3GL1#1 SS | CCTGGACTTTGACTACAAGA | Brunello Library |
|  | SH3GL1#2 SS | GGTCAGTGAGAAGGTCGGAG | Brunello Library |
|  | FTH1#1 SS | GGTGCGCCAGAACTACCACC | Brunello Library |
|  | FTH1#2 SS | CGATGATGTGGCTTTGAAGA | Brunello Library |
