# Supplementary material for: SH3GL1‐activated FTH1 inhibits ferroptosis and confers doxorubicin resistance in diffuse large B‐cell lymphoma
Source: Clin Transl Med. 2025 Mar 4;15(3):e70246. doi: 10.1002/ctm2.70246 (PMC11879899; doi:10.1002/ctm2.70246)
Supplement: Supplementary file 8 — Supporting Information [file CTM2-15-e70246-s005.docx]

| Table S4. Primer sequences used for real-time PCR | |  |
| --- | --- | --- |
| Name | Forward | Reverse |
| TFRC | AAAATCCGGTGTAGGCACAG | TTAAATGCAGGGACGAAAGG |
| NCOA4 | CCGTCACCTGGAATGTCTTAGAAG | CTGCTGAGCCTGCTGTTGAAG |
| FTH1 | CCCATTTGTGTGACTTCATTGAGAC | ATATTCCGCCAAGCCAGATTCG |
| GPX4 | CCGCTGTGGAAGTGGATGAAGATC | CTTGTCGATGAGGAACTGTGGAGAG |
| ACTIN | CCTAGAAGCATTTGCGGTGG | GAGCTACGAGCTGCCTGACG |
